# Arctiin-encapsulated DSPE-PEG bubble-like nanoparticles inhibit alveolar epithelial type 2 cell senescence to alleviate pulmonary fibrosis *via* the p38/p53/p21 pathway

**DOI:** 10.3389/fphar.2023.1141800

**Published:** 2023-03-14

**Authors:** Dian Xiong, Fei Gao, Jingbo Shao, Yueyun Pan, Song Wang, Dong Wei, Shugao Ye, Yuan Chen, Rui Chen, Bingqing Yue, Juan Li, Jingyu Chen

**Affiliations:** ^1^ Lung Transplantation Center, Department of Thoracic Surgery, Nanjing Medical University Affiliated Wuxi People’s Hospital, Wuxi, China; ^2^ Department of Emergency, Nanjing Medical University Affiliated Wuxi People’s Hospital, Wuxi, China; ^3^ Department of Emergency, Nanjing General Hospital of Nanjing Military Region, Nanjing, China; ^4^ Department of Intensive Care Medicine, Nanjing Medical University Affiliated Wuxi People’s Hospital, Wuxi, China; ^5^ Key Laboratory of Clinical Laboratory Diagnostics, Ministry of Education, College of Laboratory Medicine, Chongqing Medical University, Chongqing, China; ^6^ Department of Lung Transplantation, Second Affiliated Hospital, Zhejiang University School of Medicine, Hangzhou, China; ^7^ Department of Chemistry, Fudan University, Shanghai, China

**Keywords:** idiopathic pulmonary fibrosis, alveolar epithelial type 2 cells, cellular senescence, arctiin, nanoparticle, DSPE-PEG, p38/p53/p21 signaling axis, traditional Chinese medicine

## Abstract

**Background:** Idiopathic pulmonary fibrosis is a severe and deadly form of diffuse parenchymal lung disease and treatment options are few. Alveolar epithelial type 2 (AEC2) cell senescence is implicated in the pathogenies of IPF. A major bioactive compound from the traditional Chinese medicine *Fructus arctii*, arctiin (ARC) has robust anti-inflammatory, anti-senescence, and anti-fibrosis functions. However, the potential therapeutic effects of ARC on IPF and the underlying mechanisms involved are still unknown.

**Methods:** First of all, ARC was identified as an active ingredient by network pharmacology analysis and enrichment analysis of *F. arctii* in treating IPF. We developed ARC-encapsulated DSPE-PEG bubble-like nanoparticles (ARC@DPBNPs) to increase ARC hydrophilicity and achieve high pulmonary delivery efficiency. C57BL/6 mice were used to establish a bleomycin (BLM)-induced pulmonary fibrosis model for assessing the treatment effect of ARC@DPBNPs on lung fibrosis and the anti-senescence properties of AEC2. Meanwhile, p38/p53 signaling in AEC2 was detected in IPF lungs, BLM-induced mice, and an A549 senescence model. The effects of ARC@DPBNPs on p38/p53/p21 were assessed *in vivo* and *in vitro*.

**Results:** Pulmonary route of administration of ARC@DPBNPs protected mice against BLM-induced pulmonary fibrosis without causing significant damage to the heart, liver, spleen, or kidney. ARC@DPBNPs blocked BLM-induced AEC2 senescence *in vivo* and *in vitro*. The p38/p53/p21 signaling axis was significantly activated in the lung tissues of patients with IPF, senescent AEC2, and BLM-induced lung fibrosis. ARC@DPBNPs attenuated AEC2 senescence and pulmonary fibrosis by inhibiting the p38/p53/p21 pathway.

**Conclusion:** Our data suggest that the p38/p53/p21 signaling axis plays a pivotal role in AEC2 senescence in pulmonary fibrosis. The p38/p53/p21 signaling axis inhibition by ARC@DPBNPs provides an innovative approach to treating pulmonary fibrosis in clinical settings.

## 1 Introduction

Idiopathic pulmonary fibrosis (IPF) is a progressive, irreversible, and typically fatal lung disease characterized by airway remodeling, inflammation, alveolar destruction, and fibrosis (Wolters et al., 2018). It affects mainly male patients over the age of 60. The median survival of patients after diagnosis is 3–5 years in the absence of lung transplantation (Kropski and Blackwell, 2019); this is comparable to severe cancer disease.

The US Food and Drug Administration approved two medications to treat IPF in 2014: pirfenidone and nintedanib; however, these two therapies did not reduce IPF mortality (King and Nathan, 2015). Furthermore, frequent side effects restrict the clinical application of these antifibrotic drugs. Effective therapies with high accumulation in the lungs and low adverse side effects in other organs and tissues are urgently needed. Local administration of drugs to the lungs is a promising strategy that offers a high-effective dose and prolonged residence in the lungs (da Silva et al., 2017). Recently, nanoparticle drug delivery was used for pulmonary applications due to the increased permeability of the airway mucus layer (Ghumman et al., 2021; Han et al., 2022).

Accumulating evidence demonstrates that the pathogenesis of IPF involves accelerated aging, with senescence of alveolar epithelial type 2 (AEC2) cells playing an important role in this process (Kellogg et al., 2021; Parimon et al., 2021). A single-cell RNA-sequencing study of IPF explant tissues shows regional depletion of AEC2 cells and abnormal activation of multiple pathways related to cellular senescence (Xu et al., 2016). Previous studies (Qiu et al., 2019; Yao et al., 2021) demonstrated that therapies targeting the senescence process in AEC2s could attenuate the progression of pulmonary fibrosis. However, the pathogenesis of AEC2 senescence in IPF remains unclear. The p38 pathway is a major mitogen-activated protein kinase (MAPK) pathway initially discovered to be a mediator of inflammation and stress responses (Ono and Han, 2000). p38^MAPK^ either directly phosphorylates and activates p53 or indirectly phosphorylates p53 through its downstream kinase, casein kinase 2. It was recently discovered that the p38 pathway plays an important role in fibroblast senescence and IPF (Matsuda et al., 2020); however, the role of p38/53 signaling in AEC2 senescence remains largely unknown.

Traditional Chinese Medicine (TCM) has gradually developed an advantage in treating IPF (Zhang et al., 2021). *F. arctii*, one of the most popular TCMs, is officially listed in the Chinese Pharmacopoeia (Li et al., 2022). Arctiin (ARC), isolated from *F. arctii,* is widely investigated for its anti-inflammatory (Lee et al., 2011), antiviral (Hayashi et al., 2010), antiproliferative (Matsuzaki et al., 2008), and anticancer (Hirose et al., 2000) properties. It inhibits hydrogen peroxide-induced senescence in human dermal papilla cells *in vivo* (Bae et al., 2014), suppresses cardiac fibrosis (Li et al., 2017), and attenuates silica-induced lung injury and fibrosis (Liu J. et al., 2021). However, it is unknown whether ARC attenuates bleomycin (BLM)-induced pulmonary fibrosis and AEC2 senescence. After oral administration, most ARC is metabolized to arctigenin. The systemic delivery of ARC has several limitations, including first-pass metabolism, low accumulation in the lungs, and possible adverse side effects in other organs and tissues.

One of the main problems with the inhalation route is the thick mucus layer, which may serve as a barrier to the uptake of active drug particles (Porsio et al., 2018). In this study, our objective was to develop a nanoparticle (ARC@DPBNP) that can be administered by airway delivery to suppress AEC2 senescence for the treatment of pulmonary fibrosis. We chose 1,2-distearoyl-sn-glycero-3-phosphoethanolamine (DSPE)-polyethylene glycol (PEG) 2000 (DSPE-PEG2000) as the main amphiphilic polymer for nanocarrier formation owing to the hydrophobic nature of ARC.

## 2 Materials and methods

### 2.1 Studies in humans and animals

All animal experiments complied with the ARRIVE guidelines and were carried out in accordance with the National Research Council’s Guide for the Care and Use of Laboratory Animals. The protocol for each experiment was approved by the Research Ethics Committees of Nanjing Medical University Affiliated Wuxi People’s Hospital for Animal Research (2022–32). The study has been carried out in accordance with The Code of Ethics of the World Medical Association (Declaration of Helsinki) and was approved by the Research Ethics Committees of Nanjing Medical University Affiliated Wuxi People’s Hospital (KY22090). Informed consent was obtained from all participants.

### 2.2 Reagents

BLM was purchased from MedChemExpress (cat# HY-17565A, China), dissolved in phosphate-buffered saline (PBS) at a concentration of 10 mg/mL, and stored at −80°C in the dark. ARC was purchased from Shanghai Yuanye Bio-Technology Co., Ltd (Shanghai, China), dissolved in DMSO (Biyuntian, China) to a concentration of 200 mg/mL, and stored at −80°C in the dark. DSPE-PEG2000 was obtained from Ponsure Biological Co. (Shanghai, China). PD 169316 (an inhibitor of p38^MAPK^) was purchased from MedChemExpress (Cat# HY-10570, MCE, China), dissolved in DMSO at a concentration of 10 mM, and stored at −80°C.

### 2.3 Target fishing and network building

The chemical components of *F. arctii* were categorized using the Traditional Chinese Medicine Systems Pharmacology Database and Analysis Platform (https://tcmspw.com/tcmspsearch.php) (Ru et al., 2014), and those compounds with oral bioavailability ≥30% and drug likeness ≥0.18 were selected as potential active ingredients (Ru et al., 2014). Subsequently, the pharmacological targets of the components were identified and filtered using the Swiss Target Prediction (http://www.swisstargetprediction.ch/) (Gfeller et al., 2013) and ChEMBL databases (https://www.ebi.ac.uk/chembl/) (Gaulton et al., 2017). We searched the GeneCards (https://www.gene-(cards.org/) (Safran et al., 2010), OMIM database (https://www.omim) (Amberger et al., 2015), Drugbank database (https://www.drugbank.ca/) (Wishart et al., 2018) and TTD database (http://bidd.nus.edu.sg/group/cjttd/) (Zhou et al., 2022), using the keywords “idiopathic pulmonary fibrosis” to identify targets related to IPF. The Uniprot database (https://www.uniprot.org/) was used to standardize the target information by including the species information “*homo sapiens*.” The STRING database (http://string-db.org) (Szklarczyk et al., 2021) was used to build the protein–protein interactions (PPI) network, and the results were visualized with Cytoscape 3.9.1. Additionally, Cytoscape3.9.1 was adopted for constructing the medicine-ingredients-targets-disease network. The STRING database (http://string-db.org) was employed for Gene Ontology and Kyoto Encyclopaedia of Genes and Genome enrichment analyses, which were performed with Bioinformatics (https://www.bioinformatics.com.cn/).

### 2.4 Molecular docking

The SDF format file of the three-dimensional structure of ARC was obtained from the PubChem database (https://pubchem.ncbi.nlm.nih.gov/). The small molecule of ARC whose file was subsequently imported into ChemDraw (http://www.perkinelmer.com/tw/category/chemdraw) was processed with minimized energy and saved as a mol2 file. The initial structures of the top 8 potential targets were downloaded from the Protein Data Bank (http://www.rcsb.org/) database and were visualized using PyMoL. The target proteins were then imported into AutoDock MGLTools 1.5.6 (http://mgltools.scripps.edu/documentation/links/autodock), where they were hydrogenated, the charge calculated, and the non-polar hydrogen combination calculated. The results were stored in PDBQT format. Finally, molecular docking simulations were performed using AutoDock Vina 1.1.2, and PyMOL was used to visualize the results.

### 2.5 Preparation and characterization of ARC@DPBNPs

DSPE-PEG bubble-like nanoparticles (DPBNPs) were used as carriers to encapsulate ARC. DPBNPs were prepared as previously described (Zeng et al., 2012). Briefly, ARC (20 mg) was dissolved in CHCl_3_ (200 μL) by stirring. DSPE (50 mg, 0.06  mmoL, Ponsure Biological) and PEG 2000 (50 mg, 0.06  mmoL, Ponsure Biological) were dissolved in 200 μL chloroform. The two solutions were mixed in a 1:1 ratio to form a homogeneous organic phase. An additional 1 mL of deionized water was added to the mixture and mixed for 15 min. The solution was sonicated for 10 min using an ultrasonic mixer (Shanghai Bilon Instruments, Shanghai, China). Organic solvents were removed by rotary evaporation under reduced pressure. The deposited nanoparticle film was hydrated with deionized water to obtain a final concentration of 10 mg/mL. The nanoparticles were prepared by extrusion using Avanti mini-extruders (1 or 8 μm membranes). ARC@DPBNP absorbance properties were measured using a Hitachi UH5300 UV spectrophotometer (Tokyo, Japan), and their size was characterized using a Nano ZS zetasizer (Malvern, United Kingdom). The nanoparticle morphology was visualized using transmission electron microscopy (TEM, Tecnai G2-20).

### 2.6 Cell culture and treatment

The human AEC2 cell line (A549) was acquired from the Shanghai Cell Bank of the Chinese Academy of Sciences (Shanghai, China). A549 cells were grown in Dulbecco’s modified Eagle’s medium supplemented with 10% fetal bovine serum and 1X penicillin–streptomycin solution (Thermo Fisher Scientific, United States) at 37°C in 5% CO_2_. To create a model of cellular senescence, A549 cells were stimulated with particular doses of BLM for 72 h in Dulbecco’s modified Eagle’s medium supplemented with 5% fetal bovine serum. As described in the figure legends, cells were treated with BLM and/or various concentrations of ARC and ARC@DPBNPs.

### 2.7 Cell counting kit-8 (CCK-8) assay

In 96-well culture dishes, A549 cells were cultured for various lengths of time in a medium containing various concentrations of BLM, ARC, and ARC@DPBNPs. Each well (containing 100 L of media) received 10 µL of CCK-8 solution (Yesen, Shanghai, China), which was then cultured for 2 h at 37°C. Using a microplate reader (Type:1,510, Thermo Fisher Scientific), the absorbance of each group was determined at 450 nm (*n* = 5).

Ten microliters of CCK-8 solution (Yesen, Shanghai, China) were added to each well (containing 100 μL medium) and cultured for 2 h at 37°C. The absorbance of each group was measured at 450 nm (*n* = 5) using a microplate reader (Type:1,510, Thermo Fisher Scientific). The absorbance and number of live cells were directly proportionally related.

### 2.8 Mice and treatment

Inbred male C57BL/6J mice aged 6–8 weeks were purchased from Changzhou Cavans Animal Experiment Co., Ltd. The mice were randomly selected and divided into four groups: PBS (*n* = 9), ARC@DPBNP (*n* = 9), BLM (*n* = 9), and ARC@DPBNP + BLM (*n* = 9) groups. Pulmonary fibrosis was induced by intranasal administration of 5 mg/kg of BLM on the first day. Mice in the BLM + ARC@DPBNP groups were treated daily with ARC@DPBNPs by transnasal administration for 21 days up to the day before BLM administration. Mice in the PBS and ARC@DPBNP groups were treated with 40 μL PBS or ARC@DPBNPs by using nasal drops for 21 days. The mice were sacrificed on day 21 after treatment with BLM or PBS, and the lung, liver, spleen, heart, and kidney were collected for subsequent experiments.

### 2.9 Primary tissue sample

Human lung tissue samples were provided by the affiliated hospital. IPF tissues were collected from patients (*n* = 15; Table 1). Control lung tissues were collected from healthy adult donors (*n* = 15; Table 2) that were resected based on size incompatibility or deemed unsuitable for lung transplantation.

**TABLE 1:** Donor characteristics.

Characteristics	Mean ± SD or n% (*n* = 15)
Age (years)	42 ± 9.95
Sex: Male (%)	90.3%
Weight (kg)	67.58 ± 9.54
BMI	23.08 ± 2.68
Oxygenation index	420.5 ± 83.86
Ventilation duration (days)	16.03 ± 24.5
DCD	0%
Current smoker	19.4%
Cause of death	
TBI	32.3%
CVA or ICH	67.7%
Others	0%

Abbreviations: BMI, body mass index; CVA, cerebrovascular accident; DCD, donation after circulatory death; ICH, intracerebral hemorrhage; SD, standard deviation; TBI, traumatic brain injury.

**TABLE 2:** Baseline characteristics of the recipients.

Characteristics	Anterolateral group
	Mean ± SD or n%
Age (years)	52.80 ± 10.30
Sex: Male (%)	66.67%
Weight (kg)	60.79 ± 12.17
BMI	21.93 ± 3.98
Oxygenation index	217.36 ± 58.11
FVC (L)	1.70 ± 0.45
FEV1 (L)	1.44 ± 0.45
6MWT (m)	234.875 ± 109.85
Comorbidities	
Hypertension	3
Diabetes	1
CHD	2
Secondary pulmonary hypertension	6

Abbreviations: BMI, Body mass index; CHD, Coronary heart disease; FVC, Forced vital capacity; FEV1, Forced expiratory volume in 1 s; SD, Standard deviation; 6MWT, 6-minute walk test.

### 2.10 Histopathological assessment

Tissues were fixed in 4% paraformaldehyde for 24 h, embedded in paraffin using a paraffin embedding machine (KD-BM, Zhejiang Kedi, China), and cut into 4-μm sections using a Leica RM2016 rotary microtome. Hematoxylin–eosin and Masson’s trichrome staining were performed. According to a documented method, the Ashcroft score method was used to blindly score the level of fibrosis in the mouse lung (Porsio et al., 2018). Immunohistochemical techniques were used to identify protein expression in various tissues. The sections were deparaffinized in xylene and rehydrated in graded alcohol (absolute ethyl alcohol, 95% and 80% ethyl alcohol). Endogenous peroxidase activity was quenched with 3% aqueous hydrogen peroxide for 15 min. The tissue sections were blocked with 10% bovine serum albumin (YESEN, Shanghai, China) for 1 h at room temperature, and antigen retrieval was performed with a pressure cooker. Sections were incubated with rabbit antibody against p21 (10355-1-AP; 1:200 dilution; ProteinTech, Wuhan, China), rabbit antibody against P16-InkA (10883-1-AP; 1:1,000 dilution; ProteinTech), rabbit antibody against phospho-p38^MAPK^ (Thr180/Tyr182) (p-p38^MAPK^) (4,511; 1:800 dilution; Cell Signaling Technology, Danvers, United States), rabbit antibody against p38^MAPK^ polyclonal antibody (14064-1-AP, 1:100 dilution; ProteinTech); mouse antibody against p53 (60283-2-Ig, 1:1,000 dilution; ProteinTech) overnight at 4°C, followed by incubation for 1 h at 37°C in the dark with horseradish peroxidase-labeled secondary antibody (Gene Tech; Shanghai, China). Diaminobenzidine (Gene Tech) was used for color development. Finally, the sections were dehydrated and mounted. Collagen area and protein expression levels were independently analyzed by two investigators using ImageJ software (version 1.46; Rawak Software, Inc.).

### 2.11 Immunofluorescence assay

Immunofluorescence was performed as previously described (Ru et al., 2014). Briefly, fixed A549 cells or deparaffinized sections were stained with respective primary antibodies against p21 (1:100 dilution), p16 (1:200 dilution), p-p38^MAPK^ (Thr180/Tyr182) (1:1,600 dilution), p38^MAPK^ polyclonal antibody (1:50 dilution), and p53 (1:300 dilution) for 12 h at 4°C. The cells or tissue sections were then washed with PBS and incubated with fluorescent-conjugated secondary antibodies for 2 h at room temperature. The nuclei were visualized using 4′, 6-diamidino-2-phenylindole and dihydrochloride (4,083; Cell Signaling Technology). Immunofluorescence images were obtained using a Leica SP8 confocal microscope.

### 2.12 Quantitative analysis of hydroxyproline (HYP)

Mice were anesthetized with 2% isoflurane and subjected to cardiac perfusion to remove blood from the lungs. Approximately 100 mg of lung tissue sample (stored at −80°C) was hydrolyzed by adding 1 mL of hydrolysate and incubated at 95°C for 20 min (the samples were mixed again after 10 min of incubation). According to the manufacturer’s instructions, an HYP kit (Nanjing Jiancheng Bioengineering Institute, Nanjing, China) was used to measure the amount of HYP in the lung tissue.

### 2.13 RNA isolation and quantitative reverse transcriptase polymerase chain reaction (PCR)

Total RNA was extracted from cultured cells and lung tissues using an RNA extraction kit (R0027; Beyotime, Shanghai, China) according to the manufacturer’s instructions. cDNA was synthesized using HiScript III RT SuperMix for qRT-PCR (R323-01, Vazyme, Nanjing, China).

According to the manufacturer’s instructions, quantitative real-time PCR was performed using the ChamQ Universal SYBR qPCR master mix (Q711-02, Vazyme, Nanjing, China), and assays were performed using the Applied Biosystems 7,500 Real-time PCR Detection System (ABI, United States).

The internal standard was Glyceraldehyde-3-phosphate dehydrogenase (GADPH). Based on average CT values, mRNA levels were calculated using the GraphPad Prism 7.0 program (GraphPad Software, La Jolla, CA, United States). The following primers were designed: mouse GADPH, forward: 5′-CAT​CAC​TGC​CAC​CCA​GAA​GAC​TG-3′ and reverse: 5′-ATG​CCA​GTG​AGC​TTC​CCG​TTC​AG-3′; human GADPH, forward: 5′- GTC​TCC​TCT​GAC​TTC​AAC​AGC​G-3′ and reverse: 5′-ACC​ACC​CTG​TTG​CTG​TAG​CCA​A-3′; mouse Col1a1 forward: 5′-CCT​CAG​GGT​ATT​GCT​GGA​CAA​C-3′ and reverse: 5′-CAG​AAG​GAC​CTT​GTT​TGC​CAG​G-3′; mouse α-SMA forward: 5′- CGA​GCG​TGA​GAT​TGT​CCG​T-3′ and reverse: 5′-CCC​TGA​CAG​GAC​GTT​GTT​AG-3′; human P21 forward: 5′-AGG​TGG​ACC​TGG​AGA​CTC​TCA​G-3′ and reverse: 5′- TCC​TCT​TGG​AGA​AGA​TCA​GCC​G-3′; and human P16 forward: 5′- CTC​GTG​CTG​ATG​CTA​CTG​AGG​A-3′ and reverse: 5′- GGT​CGG​CGC​AGT​TGG​GCT​CC-3′.

### 2.14 Western blot analysis

Whole proteins from cell lysates and lung tissues were extracted using RIPA lysis solution (P0013B, Beyotime, Shanghai, China) according to the manufacturer’s instructions and measured using a BCA kit (P0009, Beyotime, Shanghai, China). Equal amounts of protein were separated on 8%–12% SDS–PAGE gels (Vazyme, Nanjing, China), blotted onto PVDF membranes (Merck Millipore, Germany), blocked with 5% nonfat milk in Tris-buffered saline with Tween-20 (TBST) (Beyotime, Shanghai, China) for 1 h, and incubated overnight at 4°C in antibody diluent (Beyotime, Shanghai, China) containing the following primary antibodies: rabbit antibody against p21 (1:1,000 dilution), rabbit antibody against p16 (1:1,000 dilution), rabbit antibody against p-p38^MAPK^ (Thr180/Tyr182) (1:1,000 dilution), rabbit antibody against p38^MAPK^ polyclonal antibody (1:1,000 dilution); mouse antibody against p53 (1:5,000 dilution); mouse antibody against GAPDH (60004-1-Ig, 1:50,000 dilution; ProteinTech); and mouse antibody against *ß*-Tubulin (10094-1-AP, 1:2,000 dilution; ProteinTech). The membranes were incubated with horseradish peroxidase-linked secondary antibody (Jackson Labs, United States) for 1 h, and protein expression was detected using ECL (Vazyme, Nanjing, China). Images were captured using the Tanon 5,200 imaging system (Shanghai, China). ImageJ software (version 2.0.0) was used for gray value analysis.

### 2.15 Measurement of senescence-associated *ß*-galactosidase (SA-β-gal)

According to the manufacturer’s instructions, an SA-gal kit (C0602, Beyotime, Shanghai, China) was used for SA-gal staining. Cells on 6-well chamber slides or frozen lung tissue sections were fixed with a fixative solution for 15 min at room temperature, rinsed three times with PBS for 5 min, and incubated with freshly prepared SA-β-gal staining solution at 37°C overnight. The cells or tissue sections were washed twice with PBS for 5 min at room temperature. The tissue sections were counterstained with fast nuclear red staining (Sigma–Aldrich) to clearly observe the alveolar structure. The images were captured using a light microscope equipped with a digital camera (Olympus Imaging System, Tokyo, Japan). At least three fields were obtained for each well of 6-well plates to calculate the SA-β-gal intensity.

### 2.16 Statistical analyses

GraphPad Prism software version 7.0 (La Jolla) was used for statistical analysis. The mean and SEM were used to express the data. A one-way analysis of variance was used to assess differences between two or more groups, and a two-tailed Student’s t-test was used to determine differences between the two groups. A *p*-value below 0.05 was accepted as significant.

## 3 Results

### 3.1 Bioinformatic analysis between fructus arctii and IPF

**FIGURE 1:**
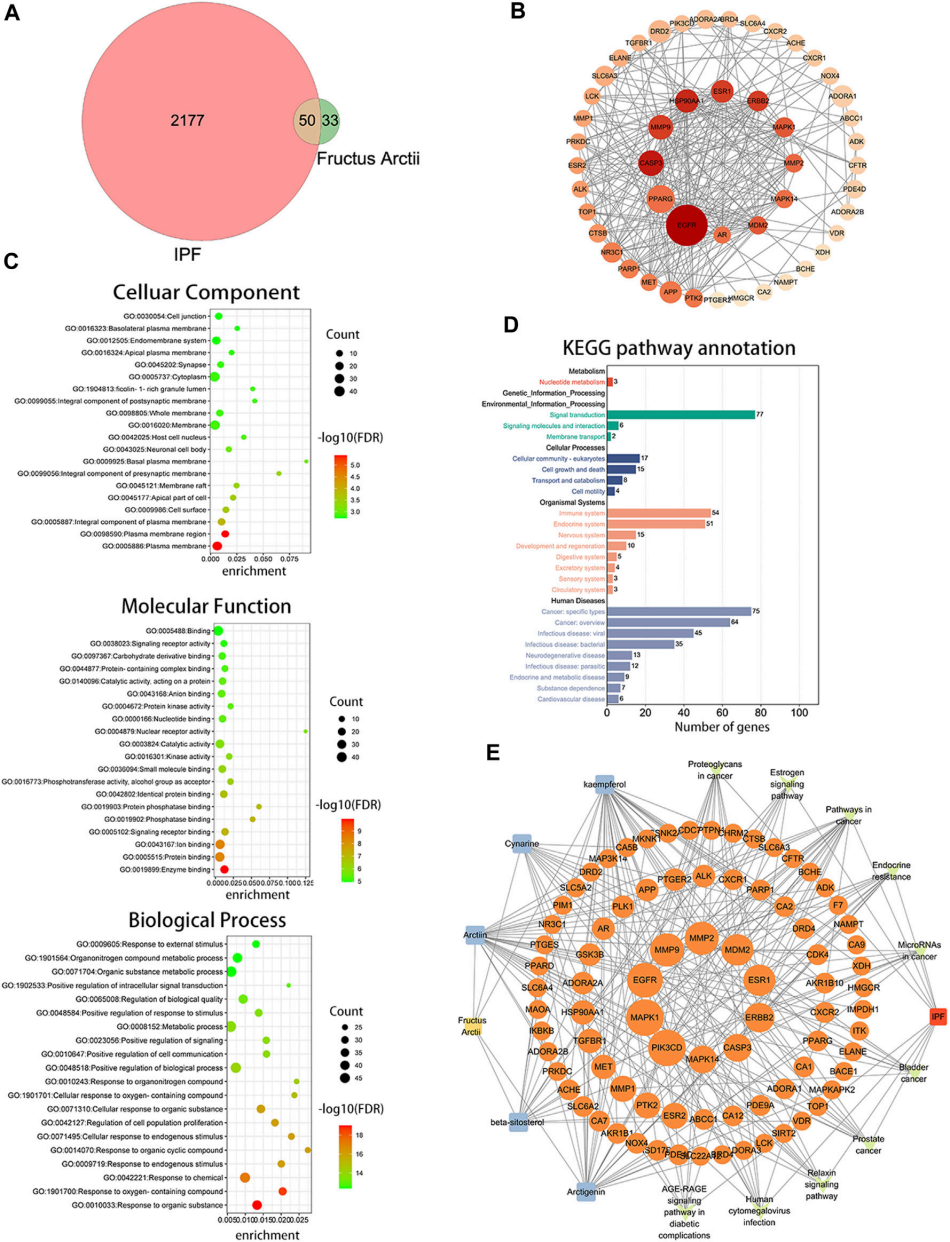
Bioinformatics analysis between *Fructus arctii* and idiopathic pulmonary fibrosis (IPF). **(A)** Venn diagram summarizing the intersected gene targets between *Fructus arctii* and the IPF. **(B)** Proteins and protein interaction (PPI) network of the 50 identified targets **(C,D)** The enrichment analysis of *Fructus arctii* targets against IPF by gene Ontology and Kyoto Encyclopaedia of Genes and Genomes analysis of intersections network **(E)** Medicine-Ingredient-Target-Disease network model of *Fructus arctii* and IPF.

**FIGURE 2:**
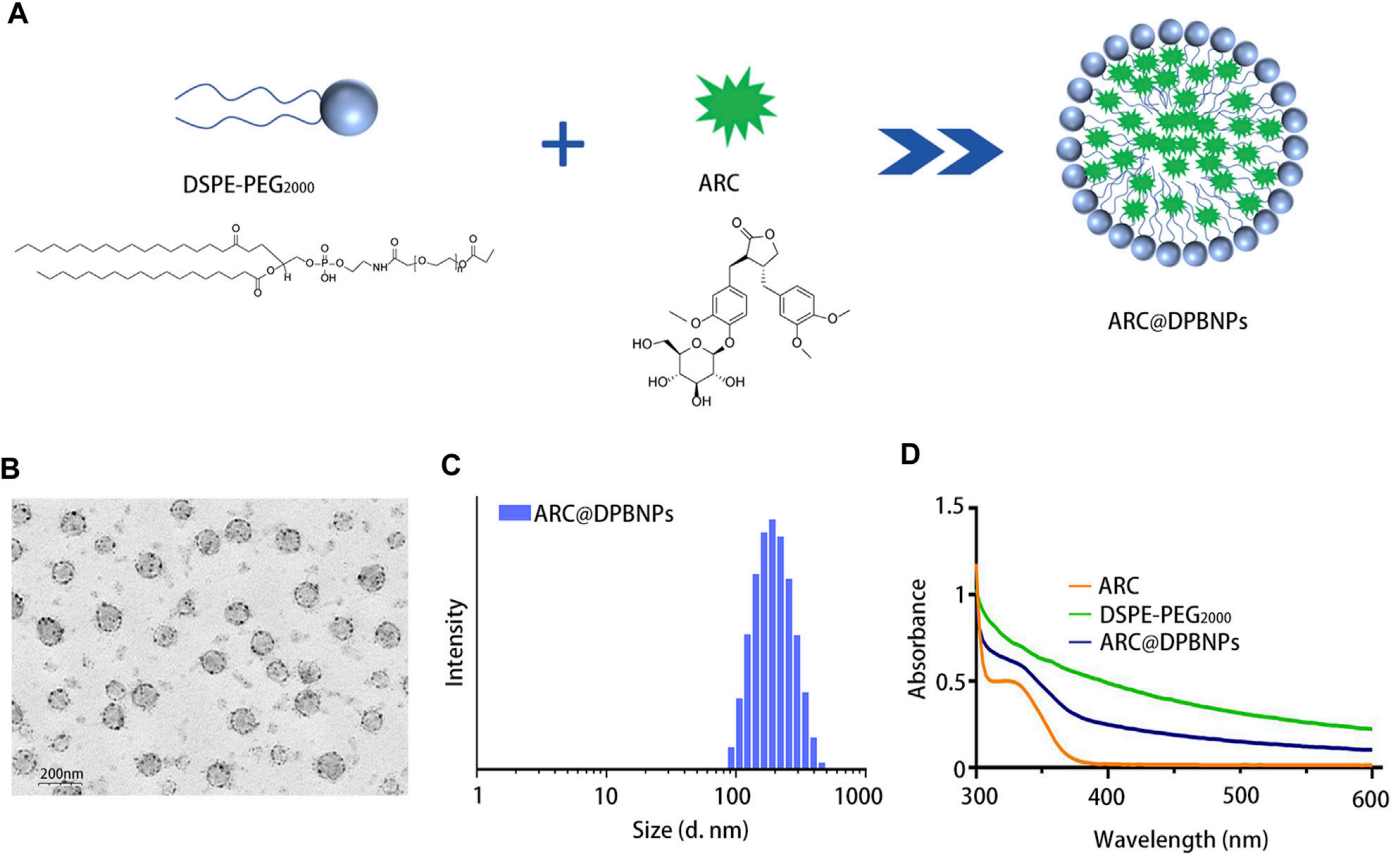
Arctiin (ARC)-encapsulated DSPE-PEG bubble-like nanoparticle (ARC@DPBNP) construction and characterization. **(A)** Chemical structures of DSPE-PEG2000 and ARC, and a schematic diagram showing ARC@DPBNP preparation. **(B)** Typical transmission electron microscopy (TEM) images of ARC@DPBNPs, scale bar = 200 nm. **(C)** Size distribution of ARC@DPBNPs according to a Nano ZS zetasizer. **(D)** Ultraviolet-visible absorption spectra of ARC, DPBNP, and ARC@DPBNP solutions.

ARC is the main bioactive compound of *F. arctii*. We initially investigated the potential mechanisms of *F. arctii* that may contribute to the treatment of IPF. In total, 144 components of *F. arctii* were preliminarily obtained from the Traditional Chinese Medicine Systems Pharmacology Database and Analysis Platform. After screening the active ingredients using the criteria of oral bioavailability ≥30% and drug likeness ≥0:18, 8 compounds were obtained. We removed the duplicated results and filtered the data using the Swiss Target Prediction and ChEMBL databases. 5 active components (ARC, beta-sitosterol, kaempferol, cynarine, and arctigenin methyl ether; Table 3) and 83 potential targets were selected for further analysis. According to methodology in previous literature, targets with a score greater than the median in the Genecards database were selected as potential IPF targets (Oh et al., 2022). After removing the duplicated results and supplementing the database with further data from OMIM, TTD, and Drugbank databases, 2227 IPF-related targets were obtained. The Venn diagram, drawn by Bioinformatics (https://www.bioinformatics.com.cn/), identified 50 potential targets, which were obtained by the intersection of *F. arctii* compound targets and IPF targets (Figure 1A). The PPI network of the 50 intersecting targets was constructed using the STRING database (http://string-db.org) and was visualized with Cytoscape 3.9.1. There were 50 nodes and 254 edges in total, the average node degree was 10.37, and the PPI enrichment *p*-value was <0.001 (Figure 2B). The top 20 screened key targets were based on three major parameters: degree, betweenness, and closeness as shown in (Supplementary Table S1).

**TABLE 3:** The top five potential active components of *Fructus Arctii*.

Mol ID	Molecule name	MW	OB%	DL
MOL000522	Arctiin	534.61	34.45	0.84
MOL000358	Beta-sitosterol	414.79	36.91	0.75
MOL000422	Kaempferol	286.25	41.88	0.24
MOL007326	Cynarine	516.49	31.76	0.68
MOL003290	Arctigenin methyl ether	386.48	52.3	0.48

Abbreviation: Mol ID, Molecule identification; MW, Molecule weight; OB, Oral bioavailability.

DL, Drug likeness.

The intersection of protein targets was subjected to Gene Ontology and Kyoto Encyclopaedia of Genes and Genome enrichment analyses. The results of the Gene Ontology (biological process) analysis showed that the intersection targets were mostly enriched in response to organic substances, response to oxygen-containing compounds, response to chemicals, etc. The molecular function was mainly involved in enzyme binding, protein binding, ion binding, signaling receptor binding, phosphatase binding, etc. Cellular component mainly included the plasma membrane, plasma membrane region, cell surface, etc. (Figure 1C). Kyoto Encyclopaedia of Genes and Genome enrichment was most involved in signal transduction, cell growth and death, immune system, and Cancer: specific types, etc. (Figure 1D). To further screen the core components and targets of *F. arctii* in treating IPF, the medicine-ingredients-targets-disease network was constructed. The results indicated that ARC, kaempferol, cynarine, beta-sitosterol, and arctigenin methyl ether regulate IPF progression by targeting 50 potential sites (Figure 1E).

### 3.2 Characterization of ARC-encapsulated DPBNPs

The molecular structures of DPBNPs and ARC and a schematic representation of the ARC@DPBNP preparation method are shown in Figure 2A. TEM analysis was performed to characterize the shape, size, and homogeneity of ARC@DPBNPs. The results showed ARC@DPBNPs had a uniform microbubble-like structure and particle size of 56.56 ± 9.78 nm (Figure 2B). The ARC@DPBNPs particles were 185.5 ± 22.3 nm according to the Nano ZS zetasizer (Figure 2C). A previous study suggested that 50–200 nm nanoparticle sizes are effective for alveolar deposition and cellular internalization after nebulization administration while avoiding clearance by alveolar macrophages (Dandekar et al., 2010). The zeta potential of ARC@DPBNPs was −31.8 ± 8.36 mV (data not shown). The prepared ARC@DPBNPs displayed the characteristic absorption peak of ARC at 324 nm. This indicated that ARC was successfully encapsulated in the DPBNPs (Figure 2D).

### 3.3 Local administration of ARC@DPBNPs mitigated pulmonary fibrosis in mice model

**FIGURE 3:**
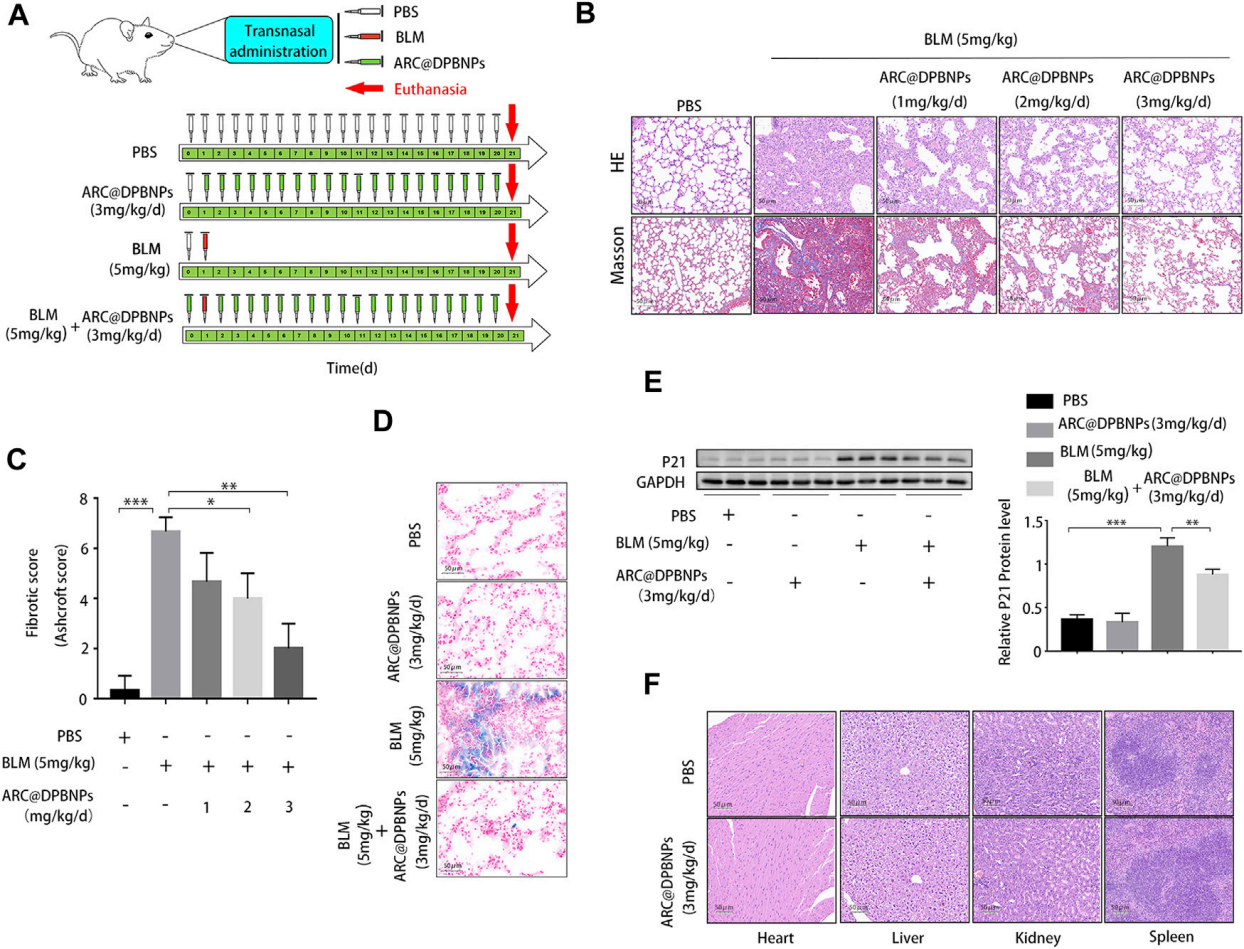
Effect and toxicity of arctiin-encapsulated DSPE-PEG bubble-like nanoparticles (ARC@DPBNPs) on pulmonary fibrosis in the mouse model **(A)** Schematic representation of the regimens of ARC@DPBNPs, phosphate-buffered saline, and bleomycin local pulmonary administration in mice **(B)** Representative photographs of lung sections of the pulmonary fibrosis in a mouse model treated with various ARC@DPBNP concentrations stained with hematoxylin–eosin (HE) and Masson’s trichrome (MA). Scale bar = 50 μm. **(C)** Fibrotic scores were analyzed. **(D)** Representative results of senescence-associated *ß*-galactosidase staining of mice lung tissues. Scale bar = 50 μm. **(E)** Senescent marker p21 in mice lung tissues measured through Western blotting. **(F)** Hematoxylin and Eosin staining of the heart, liver, spleen, and kidney performed after intrapulmonary administration of ARC@DPBNPs (3 mg/kg/d) for 21 d *p < 0.05, **p < 0.01, ***p < 0.001.

The therapeutic effects of ARC@DPBNPs were evaluated in C57BL/6 mice after BLM induction for 21 days. The mice were randomly divided into four groups: PBS-treated; ARC@DPBNP-treated; BLM-treated; and BLM + ARC@DPBNP-treated. Three doses (1, 2, and 3 mg/kg/day) were trialed to determine the optimal effective dose of ARC@DPBNPs for use in the experiment (Figure 3A). Pathological results showed that after ARC@DPBNP treatment, lung fibrosis was gradually reversed according to the dosage, compared with that in the BLM group manner (Figure 3B). The final Ashcroft fibrotic score was negatively correlated with the dose of ARC@DPBNPs (Figure 3C). Lung fibrosis was dramatically alleviated after treatment with 3 mg/kg/day ARC@DPBNPs for 21 days. SA-β-Gal-positive (blue) cell numbers increased significantly in the BLM group, and ARC@DPBNPs obviously decreased the number of SA-β-Gal-positive cells in the lung tissue of the mouse model (Figure 3D). Meanwhile, Western blot analysis showed that p21 increased significantly in the BLM group compared to that in the PBS and ARC@DPBNP groups (*p* < 0.001), whereas ARC@DPBNP treatment significantly inhibited its expression (*p* < 0.001, Figure 3E). These results indicated that ARC@DPBNPs inhibited BLM-induced cellular senescence *in vivo*. The mice administered nasal drops of ARC@DPBNPs (3 mg/kg/d) for 21 days exhibited no substantial organ damage, as evidenced by the histology of the heart, liver, spleen, and kidney (Figure 3F). Based on these results, 3 mg/kg/day was a suitable dose for subsequent animal experiments.

### 3.4 Targets prediction of ARC for the treatment of IPF

**FIGURE 4:**
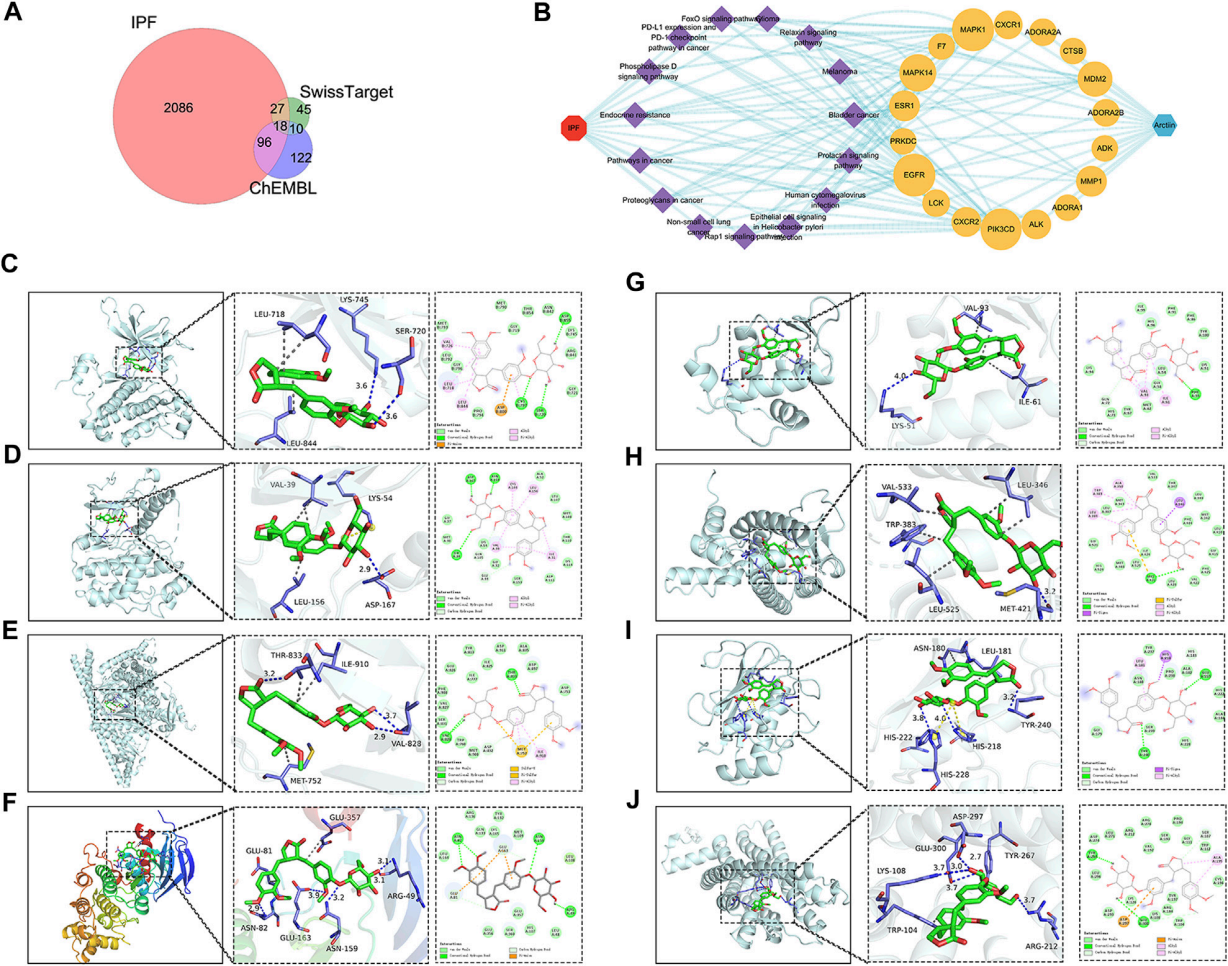
Network construction of arctiin (ARC) related to idiopathic pulmonary fibrosis (IPF) and molecular docking. **(A)** Venn diagram summarizing the intersected gene targets between ARC and the IPF. **(B)** ARC-target-pathway-IPF network. **(C–J)** ARC molecular docking and the top 8 target proteins related to IPF after visualization.

In order to clarify the novel drug target of ARC in treating IPF, network pharmacology and molecular docking analyses were employed. There were 28 genes in the intersection of the SwissTarget and ChEMBL databases, representing the most probable target for ARC. Subsequently, taking the intersection of the ARC and IPF-related targets, a total of 18 potential active targets were obtained, as shown in Figure 4A. The components-targets-pathways-disease network was then constructed to see whether ARC is combined with the top 8 core target proteins (Table 4). Figures 4C–J depicts images of the optimal docking of proteins and ARC after visualization. The docking scores for ARC with the top eight targets (EGFR, MAPK1, PIK3CD, MAPK14 (p38), MDM2, ESR1, MMP1, and CXCR2) of IPF were −7.6, −8.0, −6.5, −8.2, −6.4, −7.6, −6.6, and −6.8 kcal/mol (Supplementary Table S2), respectively. Among them, the docking of MAPK14 (p38) had the lowest binding score −8.2 kcal/mol). Given that a lower docking score represents a stronger binding affinity, and a score <−5 indicates strong binding activity (Liu X. et al., 2021), the molecular docking results indicate that ARC has a high affinity with MAPK14 (p38), which plays an essential role in cellular senescence. Figure 4G shows that the structure of ARC is linked to ARG49, ASN82, ASN159, and GLU163 in p38 through hydrogen bonds and has a hydrophobic interaction with GLU81 and GLU357.

**TABLE 4:** Top ten targets information of PPI network.

Name	Degree	Betweenness	Closeness
EGFR	16	133.059	0.630
MAPK1	16	126.667	0.630
PIK3CD	15	109.995	0.607
MAPK14	11	64.344	0.531
MDM2	9	45.223	0.515
ESR1	5	16.6286	0.459
MMP1	5	13.724	0.447
CXCR2	4	13.315	0.447

### 3.5 ARC@DPBNPs inhibited A549 cell senescence induced by BLM and had less effect on cell vitality compared to the ARC monomer

Cellular senescence caused by AEC2 plays a critical role in IPF. Targeting key regulators of cellular senescence is a potential therapeutic direction for IPF (Qiu et al., 2019; Tian et al., 2019). We assessed the effects of ARC monomer and ARC@DPBNPs on AEC2 senescence in A549 cells induced by BLM. A549 cells are commonly used as a human AEC2 model account of AEC2 as AECs are difficult to obtain and maintain in *ex vivo* culture. BLM was added at gradually increasing concentrations (0–30 μg/mL) to 5% fetal bovine serum culture medium for 72 h to stimulate A549 cells to generate the cellular senescence model. The percentage of SA-β-gal-positive cells gradually increased with higher concentrations of BLM (Supplementary Figure S1A). Furthermore, the relative mRNA and protein levels of senescence-related markers (p21 and p16) increased in a dose-dependent manner in stimulated A549 cells (Supplementary Figures S1B, S1C).

**FIGURE 5:**
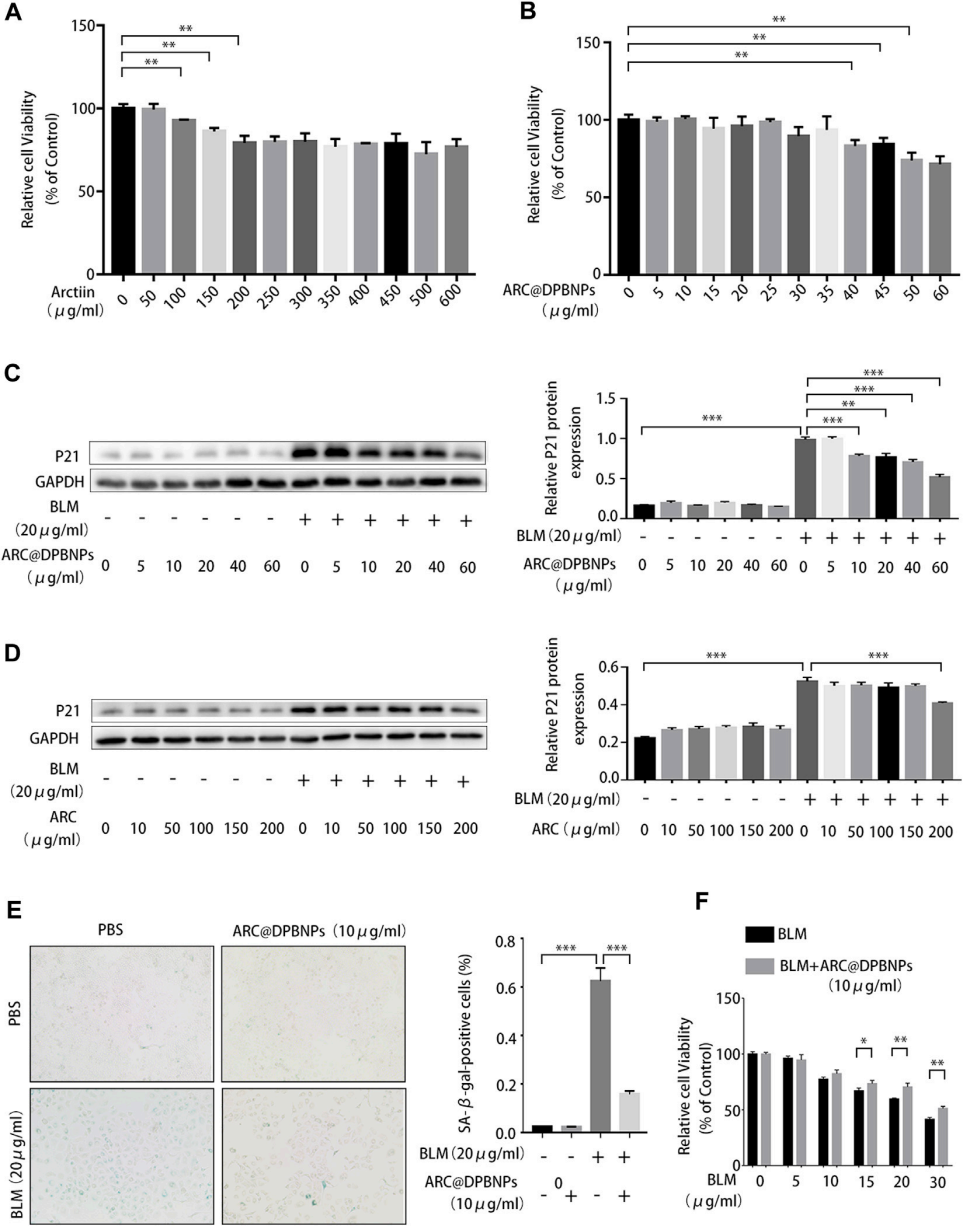
Arctiin (ARC)-encapsulated DSPE-PEG bubble-like nanoparticles (ARC@DPBNPs) inhibit A549 cell senescence induced by bleomycin (BLM) with less effect on cell vitality compared to the arctiin monomer **(A,B)** Cell counting kit-8 (CCK-8) assays were performed after A549 cells were stimulated with ARC (0–600 μg/mL) or ARC@DPBNPs (0–60 μg/mL) for 72 h **(C,D)** ARC@DPBNP (0–60 μg/mL) or ARC (0–600 μg/mL) was used to cure A549 cell senescence induced by BLM. **(E)** Senescence-associated *ß*-galactosidase (SA-β-gal) staining was used to identify cellular senescence (original magnification = ×200) **(F)** Decreased A549 proliferation stimulated with 20 μg/mL or 30 μg/mL bleomycin can be partially reversed by ARC@DPBNPs. *p < 0.05, **p < 0.01, ***p < 0.001.1.

The CCK-8 assay was performed after A549 cells were stimulated with ARC (0–600 μg/mL) or ARC@DPBNPs (0–60 μg/mL) for 72 h. The proliferation of A549 cells was reduced by 7.25% (*p* < 0.01) with the addition of ARC at 100 μg/mL, and the value of OD450 was significantly reduced (16.8%, *p* < 0.01) at 40 μg/mL ARC@DPBNP compared to that in the control group (Figures 5A,B). We then treated senescent A549 cells with different concentrations of ARC and ARC@DPBNPs. P21 levels increased significantly in both groups in response to 20 μg/mL BLM. As shown in Figures 5C,D, the effective concentration of ARC for A549 senescence was above 200 μg/mL, whereas the minimal effective dose of ARC@DPBNPs was 10 μg/mL. A 10 μg/mL concentration of ARC@DPBNPs was selected for further detection. The intensity of positive SA-β-gal staining decreased significantly in the ARC@DPBNPs + BLM group compared to the BLM group (*p* < 0.001, Figure 2E). The decrease in A549 proliferation stimulated with 20 or 30 μg/mL BLM was partially reversed by ARC@DPBNPs (Figure 5F).

Overall, these results indicate that ARC@DPBNPs substantially inhibited A549 senescence without cell toxicity. Meanwhile, the effective dose required for the ARC monomer to downregulate p21 induced by BLM in A549 cells was beyond the toxic dose.

### 3.6 Senescent markers and the p38/p53/p21 pathway were significantly increased in IPF, BLM-induced A549 cell senescence, and pulmonary fibrosis

**FIGURE 6:**
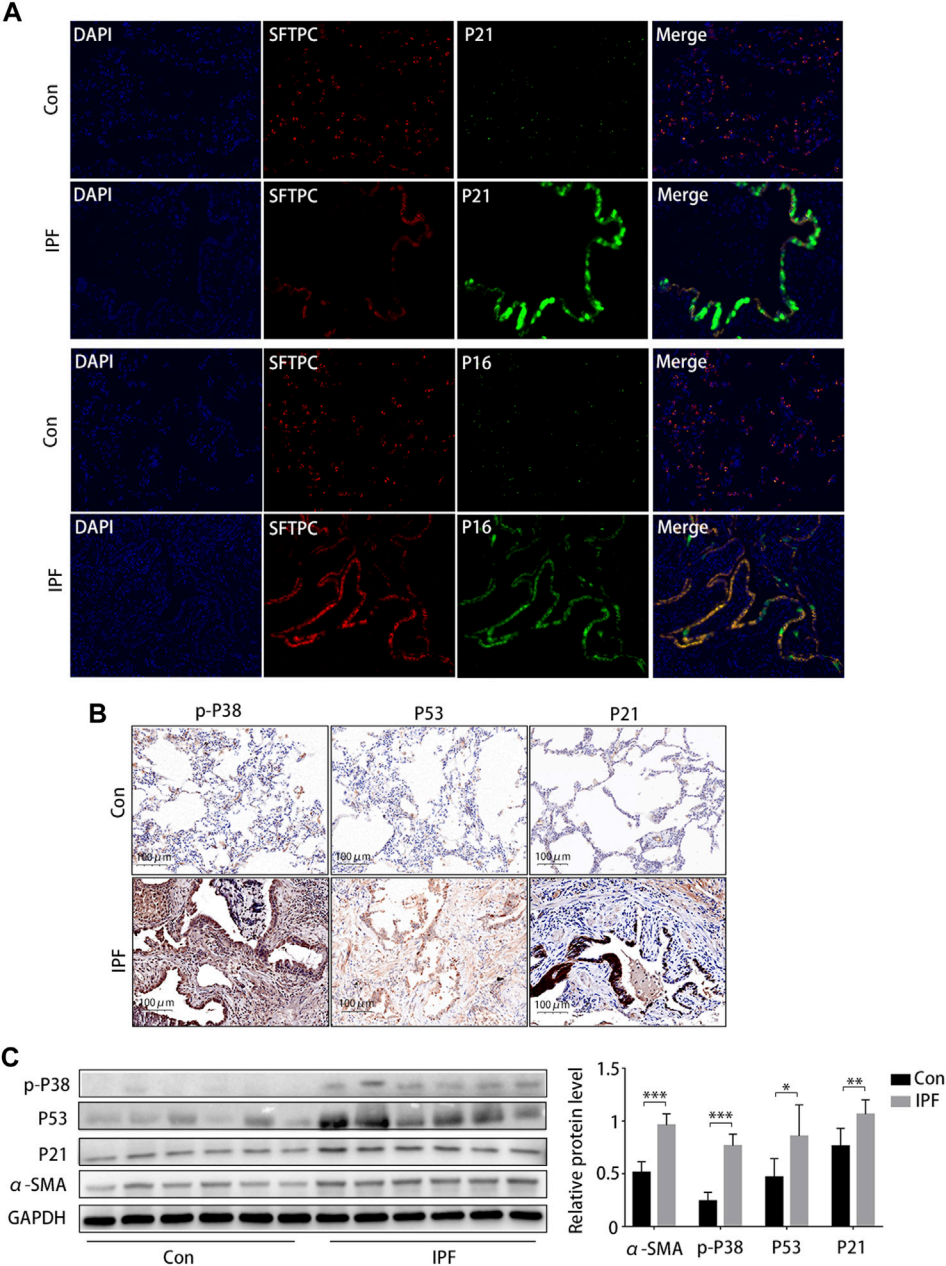
Activated p38/p53/p21 pathway in lung tissue from patients with idiopathic pulmonary fibrosis (IPF). **(A)** Immunofluorescence staining of SFTPC (an AEC2-specific marker, red), p21 (green), and p16 (green) to confirm the expression of increased senescent markers in patients with IPF (original magnification, ×400). **(B)** Representative results of immunohistochemical staining for p-p38, p53, and p21 in the lungs of patients with IPF and normal lungs (scale bar, 50 μm). **(C)** Western blot of p-p38, p53, and p21 expression. *p < 0.05, **p < 0.01, ***p < 0.001.

Staining (HE and MA) showed notable damage to pulmonary tissue and collagen deposition in patients with IPF compared to the control group, and immunohistochemical results suggested that p16 and p21 were overexpressed in the lung tissue of patients with IPF (Supplementary Figure S2). Subsequently, the Immunofluorescence staining results showed that p21 and p16 were significantly upregulated in AEC2 (SFTPC positive) cells in IPF lung tissues (Figure 6A). The immunohistochemical results (Figure 6B) and Western blotting results (Figure 6C) showed that the protein expression of α-SMA, p-p38^MAPK^, p53, and p21 was significantly increased.

**FIGURE 7:**
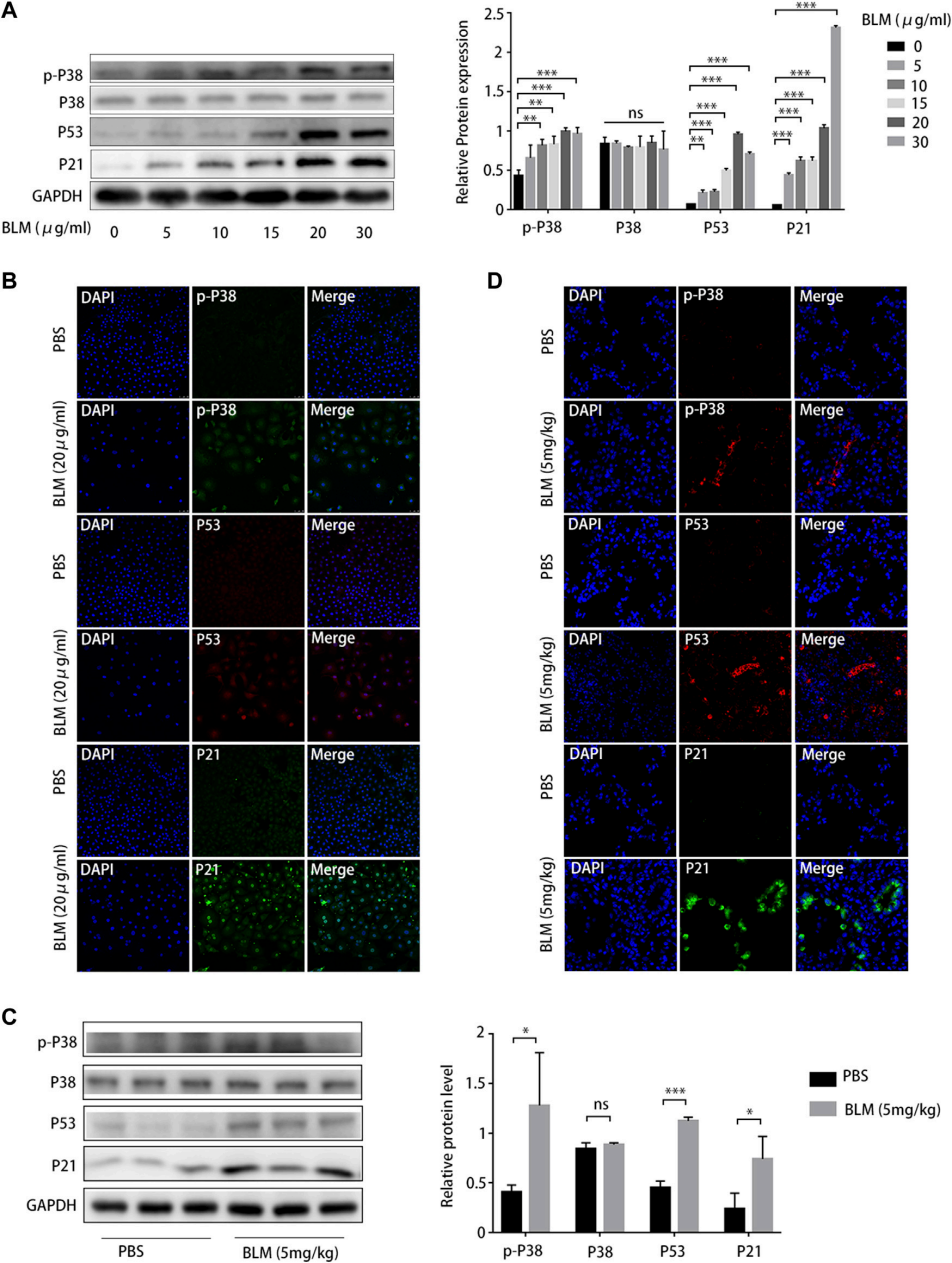
P38/P53/P21 pathway is activated in A549 senescent cells and bleomycin (BLM)-induced pulmonary fibrosis mouse model. **(A)** Western blot of p-p38, p53, and p21 levels in A549 cells treated with different BLM concentrations (0–30 μg/mL). **(B)** Immunofluorescence staining of p-p38, p53, and p21 in A549 cells treated with phosphate-buffered saline (PBS) or BLM (20 μg/mL). **(C)** Western blot of p-p38, p53, and p21 expression in lung tissue from mouse models treated with PBS or BLM (5 mg/kg) for 21 d. **(D)** Immunofluorescence staining of p-p38, p53, and p21 in lung sections of a mouse model treated with PBS or BLM (5 mg/kg) for 21 d. *p < 0.05, **p < 0.01, ***p < 0.001; ns: not statistically significant.

As cellular senescence markers were overexpressed in IPF lung tissues and the p38/p53/p21 pathway was activated, we investigated whether the p38/p53/p21 pathway participates in the senescence of A549 cells and BLM-induced pulmonary fibrosis. The p38/p53/p21 pathway was gradually activated as the BLM concentration increased (Figure 7A). The level of p-p38^MAPK^, p53, and p21 increased markedly at 20 mg/mL BLM. Meanwhile, the total p38 did not change significantly (Figure 7A). This followed immunofluorescence analysis of the p38/p53/p21 pathway in A549 cells, showing that the fluorescence intensity of p-p38^MAPK^, p53, and p21 increased markedly after 20 mg/mL BLM treatment (Figure 7B). Western blotting showed that p-p38^MAPK^, p53, and p21 protein levels were significantly upregulated in the mouse model of BLM-induced pulmonary fibrosis, and no changes in total p38 protein expression were observed (Figure 7C). The fluorescence intensity of p-p38^MAPK^, p53, and p21 increased significantly in the mouse model of BLM-induced pulmonary fibrosis compared to that in the PBS group (Figure 7D). This correlates with the results of the *in vitro* experiment. Together, these findings suggest that the p38/p53/p21 pathway is significantly activated in IPF, BLM-induced AEC2 senescence, and pulmonary fibrosis.

### 3.7 ARC@DPBNPs attenuated A549 senescence by inhibiting the p38/p53/p21 pathway

Because p38/p53/p21 plays an important role in AEC2 senescence and pulmonary fibrosis, the effect of ARC@DPBNPs on the p38/p53/p21 signaling pathway was further investigated *in vitro*. PD169316, a specific p38 inhibitor, selectively inhibits the kinase activity of phosphorylated p38 (Chen et al., 2020).

**FIGURE 8:**
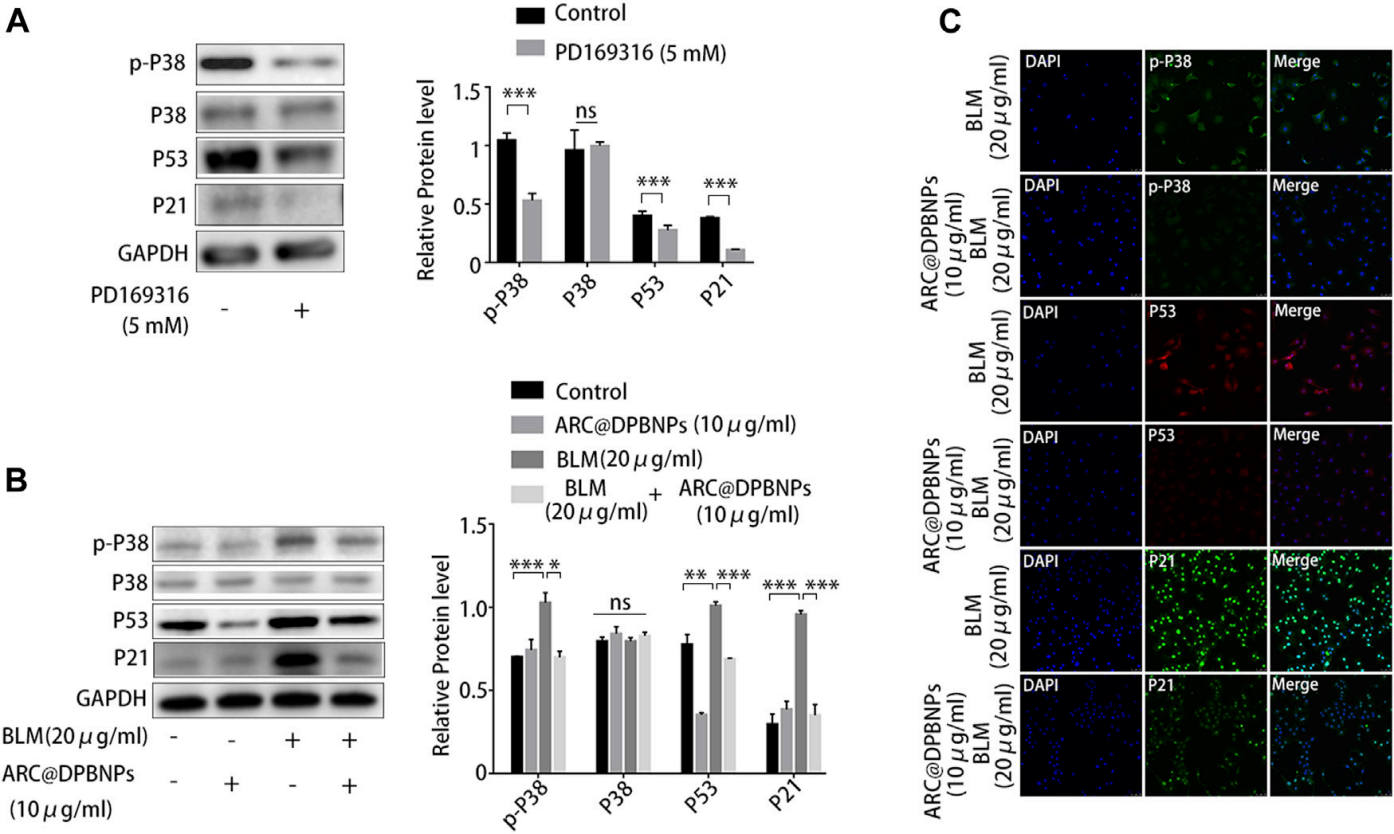
Arctiin-encapsulated DSPE-PEG bubble-like nanoparticles (ARC@DPBNPs) attenuated A549 senescence inhibiting the p38/p53/p21 pathway. **(A)** Western blot showing PD169316 selectively inhibits the kinase activity of phosphorylated p38 and p38/p53/p21 pathway activation. **(B)** Western blot showing that ARC@DPBNPs significantly attenuated A549 senescence by downregulating p-p38^MAPK^, p53, and p21 expression. **(C)** Immunofluorescence staining of p-p38^MAPK^, p53, and p21 in A549 cells treated with PBS, ARC@DPBNPs (10 μg/mL), bleomycin (BLM) (20 μg/mL), and BLM (20 μg/mL) + ARC@DPBNPs (10 μg/mL) respectively. *p < 0.05, **p < 0.01, ***p < 0.001; ns: not significant.

PD169316 added at a concentration of 5 Mm significantly inhibited p38 phosphorylation and downregulated p53 and p21 in A549 (Figure 8A). Western blotting showed that 10 μg/mL ARC@DPBNP inhibited the protein expression of p-p38^MAPK^, p53, and p21 (which was increased by 20 μg/mL BLM) in A549 cells (Figure 8B). These observations were validated through immunofluorescence staining (Figure 8C). The fluorescence intensity of p-p38^MAPK^, p53, and p21 decreased markedly in BLM-induced A549 senescence models after ARC@DPBNP treatment. Furthermore, p-p38^MAPK^, P53, and P21 decreased markedly in mice treated with a combination of BLM and ARC@DPBNPs compared with those in the BLM group (Figure 8C). This shows that ARC@DPBNPs exert anti-senescent and antifibrotic effects by suppressing p38/p53/p21 pathway activation *in vitro*.

### 3.8 ARC@DPBNPs attenuated pulmonary fibrosis and suppress AEC2 senescence by inhibiting the p38/p53/p21 pathway

**FIGURE 9:**
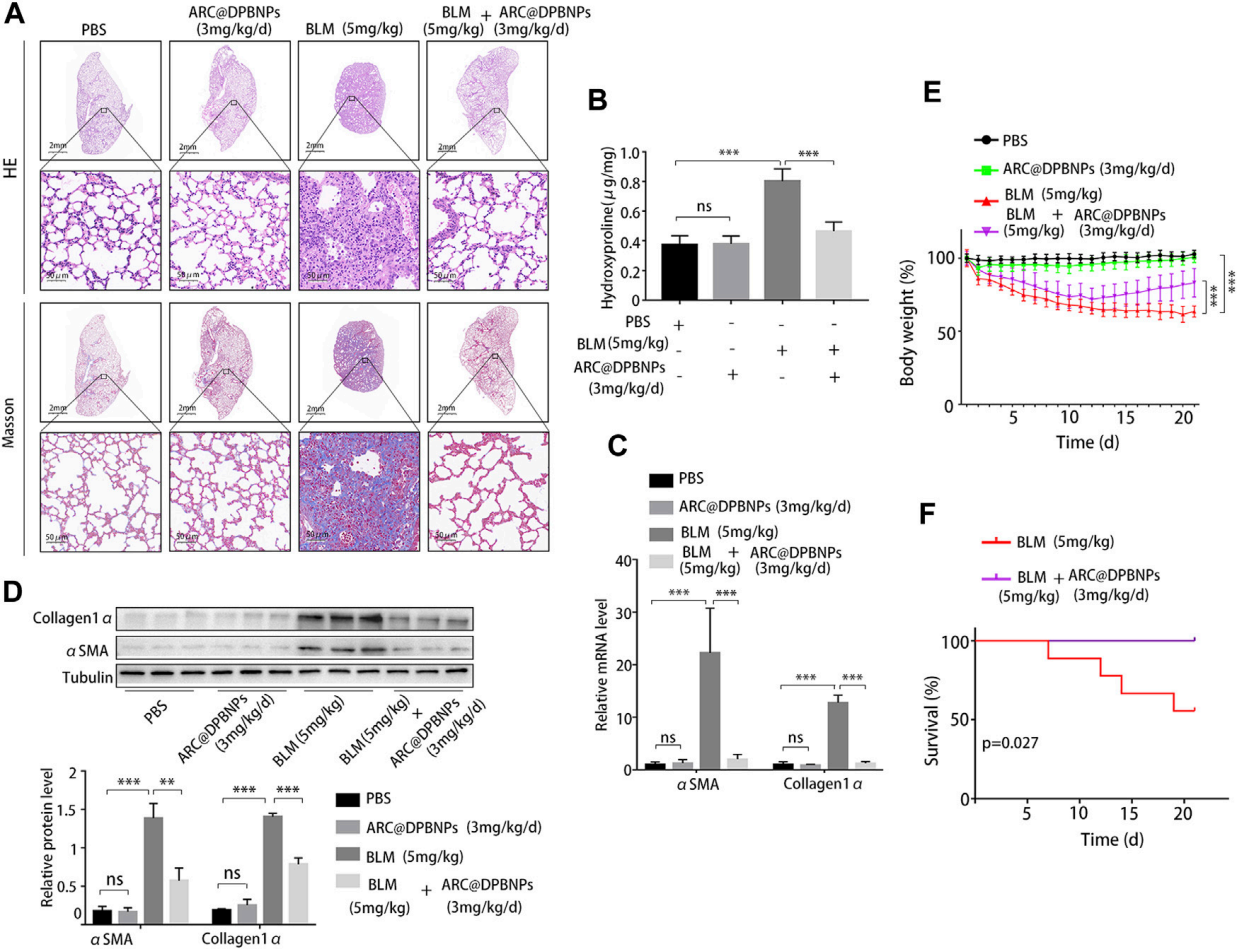
Arctiin-encapsulated DSPE-PEG bubble-like nanoparticle (ARC@DPBNP) treatment significantly alleviated pathological changes and improved the outcome of the bleomycin (BLM) mouse model. **(A)** Typical images of lung sections with hematoxylin–eosin and Masson’s trichrome staining showing that ARC@DPBNPs (3 mg/kg/d) significantly alleviated the disturbed pulmonary structure of the BLM-induced pulmonary fibrosis mouse model. No excessive pulmonary damage was observed. **(B)** Hydroxyproline content in the mouse lungs was measured on day 21. **(C)** Relative mRNA expression and **(D)** relative protein levels of lung fibrosis markers (α-SMA and collagen1α) in the lungs of mouse models on day 21. **(E)** Body weight was measured daily, and the variations (in grams) from the starting point are shown. **(F)** Survival curve of the BLM and BLM + ARC@DPBNP groups (*n* = 9 for each group). *p < 0.05, **p < 0.01, ***p < 0.001.

Based on the above results, 3 mg/kg/day of ARC@DPBNPs was chosen for subsequent experiments. Mice aged 6–8 weeks were divided into four groups, with 9 mice in each group. The lung tissues showed interstitial inflammation, fibrotic thickening of the alveolar walls, ablation of the alveolar space, and severe interstitial fibrosis after the BLM administration. Treatment with ARC@DPBNPs led to a noticeable alleviation of pathological lung lesions (Figure 9A). HYP content in the lungs of mouse models was measured as an index of collagen accumulation. As expected, the concentration of HYP in the BLM group was significantly higher than that in the PBS and ARC@DPBNP groups (Figure 9B). Collagen1α and α-SMA are commonly used as biomarkers for pulmonary fibrosis. The mRNA and protein levels of these markers in the BLM group were significantly increased compared with those in the PBS and ARC@DPBNP groups (Figures 9C,D). Mice in the BLM group gradually lost weight during the 21-day experimental period, whereas those in the BLM + ARC@DPBNP group slowly decreased body weight, and the trend reversed more quickly than the BLM group (*p* < 0.001, Figure 9E). Four mice (44.4%) died owing to the severity of the model during the 21-day experiment period, but the survival rate increased significantly after ARC@DPBNP treatment (*p* = 0.027, Figure 9F).

**FIGURE 10:**
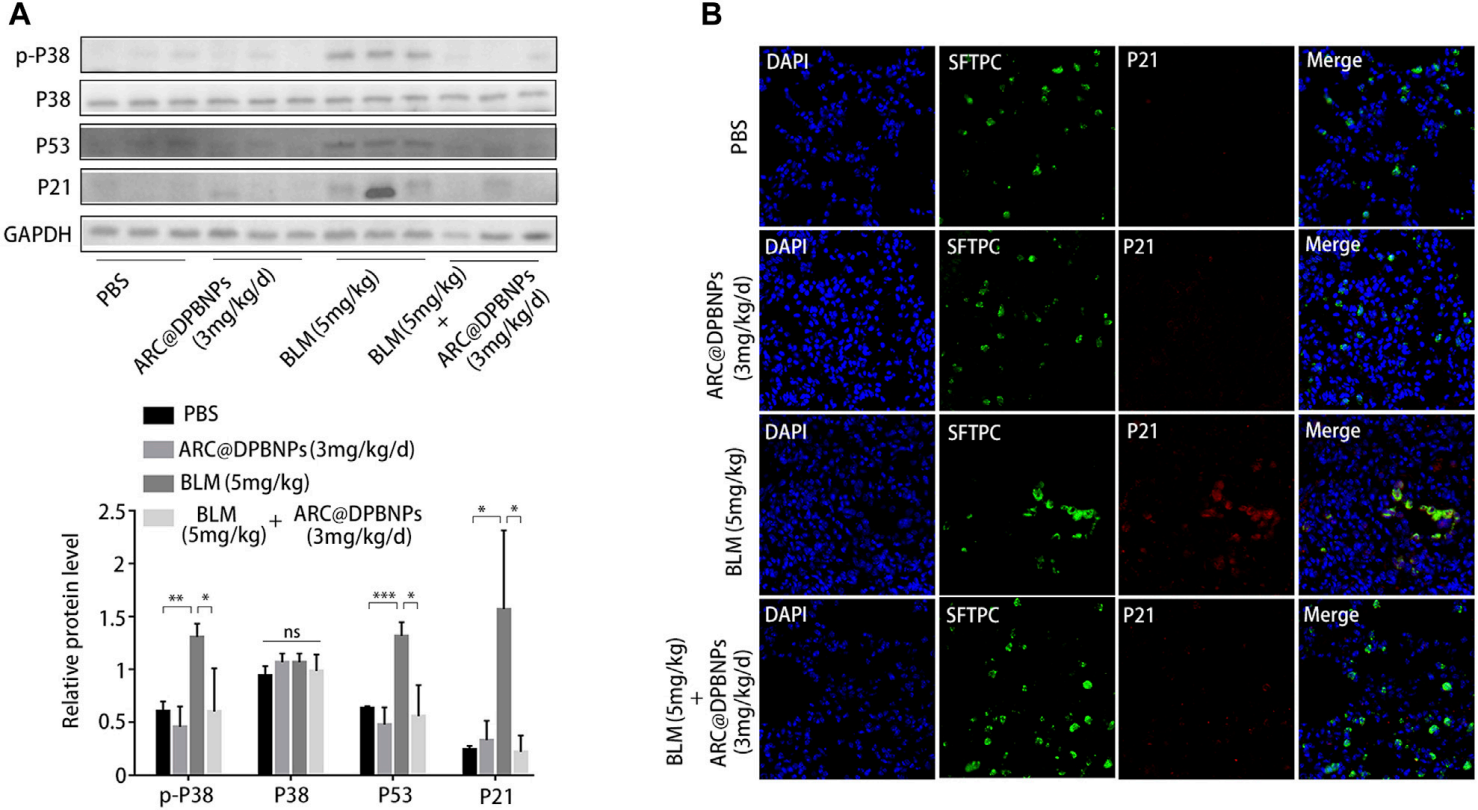
Arctiin-encapsulated DSPE-PEG bubble-like nanoparticles (ARC@DPBNPs) suppress AEC2 senescence by inhibiting the p38/p53/p21 pathway. **(A)** Western blot of p-p38MAPK, p53, and p21 expression in lung tissue of mouse models treated with phosphate-buffered saline, ARC@DPBNPs (3 mg/kg/mL), bleomycin (BLM) (5 mg/kg), and BLM (5 mg/kg) + ARC@DPBNPs (3 mg/kg/mL) respectively. **(B)** Immunofluorescence staining of SFTPC (an AEC2‐specific marker, green) and p21 (red) was performed to confirm that senescent markers were expressed primarily in the AECs (original magnification = ×400). *p < 0.05, **p < 0.01, ***p < 0.001; ns: not significant.

Western blot results showed that p-p38MAPK (*p* < 0.05), P53 (*p* < 0.05), and P21 (*p* < 0.05) decreased markedly in mice treated with a combination of BLM and ARC@DPBNPs compared with those in the BLM group (Figure 10A). Meanwhile, the expression of p21 in AEC2 cells was significantly decreased in the BLM + ARC@DPBNP group compared with that in the BLM group (Figure 10B).

Taken together, these data confirmed that ARC@DPBNPs alleviated BLM-induced pulmonary fibrosis and suppressed cellular senescence by inhibiting the p38/p53/p21 pathway.

## 4 Discussion

**FIGURE 11:**
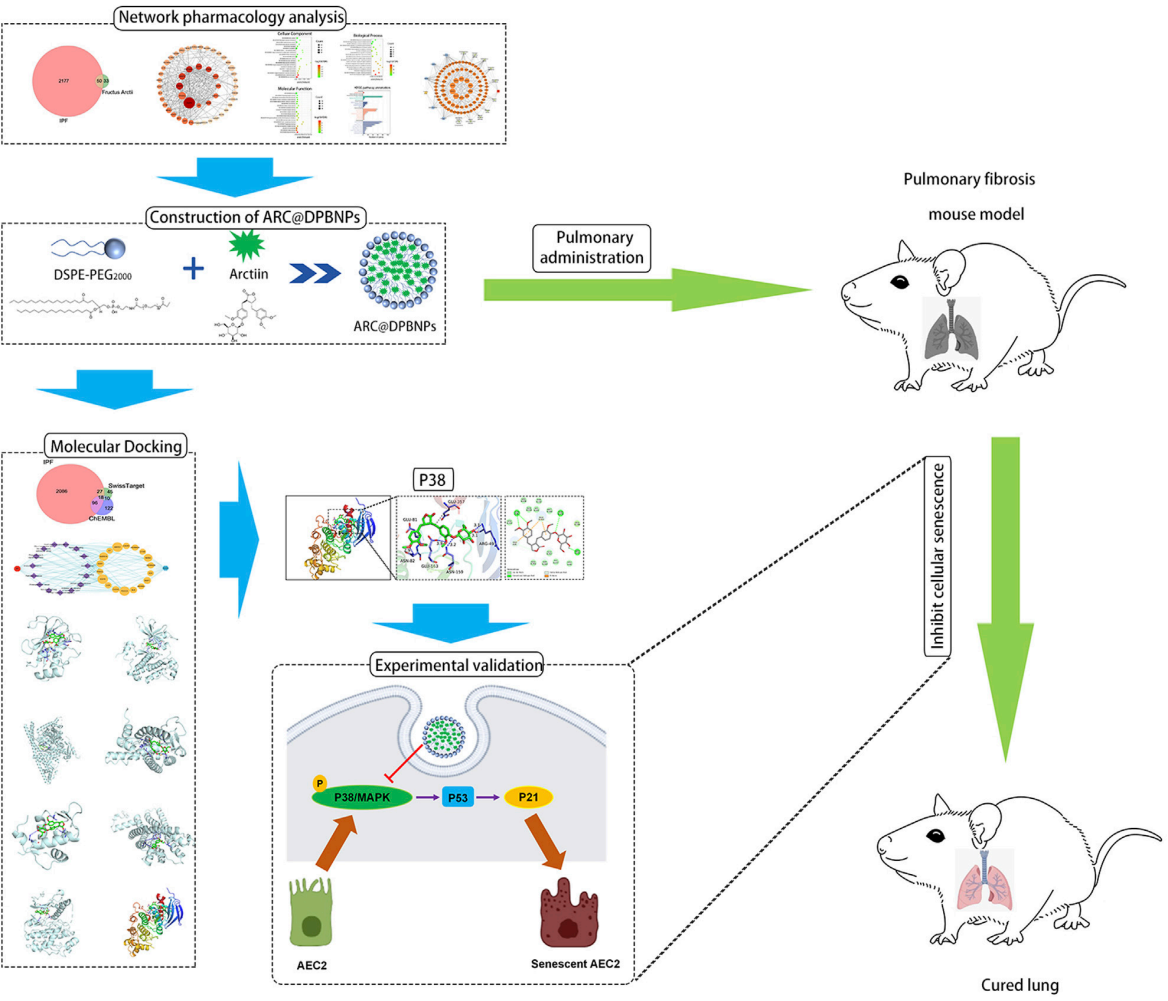
The workflow to identify the proposed mechanism showing that arctiin (ARC)-encapsulated DSPE-PEG bubble-like nanoparticles (ARC@DPBNPs) inhibit senescence of AEC2 to alleviate pulmonary fibrosis *via* the p38/p53/p21 pathway.

In this study, DPBNPs was first used to encapsulate ARC as a pulmonary drug delivery system. DPBNPs dramatically improved the hydrophilicity and deliverability of ARC. The effects of ARC@DPBNPs on BLM-induced pulmonary fibrosis and senescence were assessed *in vivo* and *in vitro*. The result indicated that pulmonary administration ARC@DPBNPs significantly reduce BLM-induced lung fibrosis and inhibited AEC2 senescence. Systematic network pharmacology and molecular docking were used to predict the mechanisms of ARC in alleviating pulmonary fibrosis, which suggested that ARC has a high affinity with p38. We confirmed the p38/p53/p21 signaling axis was significantly activated in the lung tissues of patients with IPF, senescent AEC2, and BLM-induced lung fibrosis. Our results further defined that ARC@DPBNPs attenuated AEC2 senescence and pulmonary fibrosis by inhibiting the p38/p53/p21 pathway. This study offers the first concrete proof that ARC@DPBNPs reduce BLM-induced lung fibrosis and AEC2 senescence. The workflow and proposed mechanism of ARC@DPBNPs alleviate pulmonary fibrosis are shown in Figure 11.

TCM has a long history in the treatment of pulmonary fibrosis through a multi-level and multi-targeted approach (Zhang et al., 2021). ARC is the active ingredient of biennial dried ripe burdock (*Arctium lappa* L.) belonging to the Asteraceae family. *In vitro* incubation of ARC with different cells showed anti-necroptosis and anti-oxidative effects (Bae et al., 2014; Chen et al., 2020). However, the systemic administration of ARC has several limitations, including the first-pass elimination effect and internal metabolism, causing decreased efficacy and increased side effects. DSPE-PEG is an amphiphilic material that constitutes the main structural framework of the DPBNP wall; it contains a hydrophilic PEG polymer as the external shell and a hydrophobic DSPE core. PEG modifies the surface of nanocarriers to increase the permeability of the airway mucous layer and facilitates efficient pulmonary drug delivery (Rommasi and Esfandiari, 2021; D’Souza and Shegokar, 2016). The DPBNP system has been used as a nanocarrier for hydrophobic agents (Zeng et al., 2012; Zariwala et al., 2014; Wang et al., 2021). This study used nanoparticles to load the hydrophobic ARC, thereby improving the water solubility and biocompatibility of ARC. The observed particle size of ARC@DPBNP under TEM (56.56 ± 9.78 nm) was smaller than the Nano ZS zetasizer data (185.5 ± 22.3 nm), which correlates with the results of a previous study (Zeng et al., 2012). This might be mainly attributed to the thicker PEG hydrogel layer encapsulating the surface of the nanoparticles that can be detected by the Nano ZS zetasizer. The thicker PEG hydrogel layer and smaller particle size contribute to the high-water solubility and mucus penetration ability (Han et al., 2022).

This study demonstrated that ARC inhibited BLM-induced senescence in A549 cells, and DEPG modification enhanced the biological activity of ARC@DPBNPs. ARC suppressed AEC2 senescence at 200 μg/mL *in vitro* while affecting cell viability at 100 μg/mL. However, ARC@DPBNPs had a therapeutic effect on A549 cell senescence at a lower dose and no obvious effect on cell viability. Meanwhile, intratracheal administration of ARC@DPBNPs significantly reversed BLM-induced pulmonary fibrosis without obvious side effects.

Accumulating evidence indicates that IPF is an aging-related disease, and senescent epithelial cells secrete high levels of growth factors, cytokines, chemokines, and matrix metalloproteinases that promote abnormal and persistent fibroblast activation and remodeling (Mora et al., 2017). Cellular senescence is characterized by prolonged and essentially irreversible cell-cycle arrest due to the upregulation of cell-cycle inhibitors (including p53/p21 and/or p16) and the high activity of SA-β-gal. This study confirmed AEC2 senescence in IPF lungs, BLM-induced mice, and A549 cells. ARC@DPBNP treatment significantly decreased BLM-induced p38/p53/p21 and *ß*-galactosidase levels.

P38^MAPK^ is an evolutionarily conserved serine/threonine MAPK that links extracellular signals to intracellular machinery to regulate various cellular processes, including cell apoptosis, cell cycle, and migration (Kim et al., 2017). P38 signaling is involved in the pathogenesis of pulmonary fibrosis (Matsuda et al., 2020). P38 inhibitors (SB239063 and FR-167653) alleviate BLM-induced pulmonary fibrosis (Underwood et al., 2000; Matsuoka et al., 2002). Macrophage-specific loss of function of forkhead box M1 (a p38 signaling pathway inhibitor) exacerbates BLM-induced pulmonary fibrosis (Goda et al., 2020). Furthermore, the activity of p38 and its related molecules increases in AEC2 in BLM-induced pulmonary fibrosis in mice. P53 is considered one of the most important downstream targets of p38^MAPK^. P38^MAPK^ activation promotes p53 function, and inhibition of p38^MAPK^ prevents the induction of p53 transcriptional activity (Sanchez-Prieto et al., 2000). This study observed increased p38/p53/p21 signaling in IPF AEC2 and BLM-induced models, which is consistent with the results of previous studies. SwissTarget and ChEMBL showed that p38 was one of the top 15 targets of ARC. Taken together, we hypothesized that ARC@DPBNPs inhibit AEC2 by blocking the p38 pathway.

This study had several limitations. First, we did not investigate the tissue biodistribution of ARC@DPBNPs. We also observed that the antifibrotic effects of ARC@DPBNPs at 3 mg/kg were significantly lower than those observed in other *in vivo* experiments (Li et al., 2017). In addition, we did not compare the biological activity and side effects of ARC@DPBNPs with those of the ARC monomer in a mouse model. The experiment was difficult to perform owing to the different routes of administration, bioavailability, and pharmacokinetics. Moreover, the safe dose range of ARC@DPBNP was not determined, but hematoxylin–eosin staining showed that there were no significant histological changes in the heart, liver, kidney, and spleen of the ARC@DPBNP group compared to those in the PBS group.

In conclusion, the DPBNP delivery system significantly increased the water solubility and biocompatibility of ARC. AEC2 senescence controlled by the p38-p53 pathway is a characteristic of lung fibrosis, and intratracheal injection of ARC@DPBNPs attenuated BLM-induced pulmonary fibrosis in male C57BL/6 mice. ARC@DPBNPs blocked BLM-induced AEC senescence *in vivo* and *in vitro*, probably through regulation of the p38/p53/p21 signaling pathway. These findings could guide the exploration of the therapeutic potential of ARC@DPBNPs in patients with IPF.

## Data Availability

The original contributions presented in the study are included in the article/Supplementary Materials, further inquiries can be directed to the corresponding authors.

## Abbreviations

AEC2, Alveolar epithelial type 2 cells; ARC, Arctiin; ARC@DPBNPs, ARC@DSPE-PEG bubble-like nanoparticles; BLM, Bleomycin; DSPE, 1,2-distearoyl-sn-glycero-3-phosphoethanolamine; HYP, Hydroxyproline; IPF, Idiopathic pulmonary fibrosis; MAPK, Mitogen-activated protein kinase; PEG 2000, Polyethylene glycol 2000; TCM, Traditional Chinese Medicine; SA-β-gal, Senescence-associated beta galactosidase; PBS, Phosphate-buffered saline; PCR, Polymerase chain reaction.
