# Supplementary material for: Arctiin-encapsulated DSPE-PEG bubble-like nanoparticles inhibit alveolar epithelial type 2 cell senescence to alleviate pulmonary fibrosis via the p38/p53/p21 pathway
Source: Front Pharmacol. 2023 Mar 14;14:1141800. doi: 10.3389/fphar.2023.1141800 (PMC10043219; doi:10.3389/fphar.2023.1141800)
Supplement: Supplementary file 1 [file Presentation1.pdf]

Supplementary Information

Figure S1. Bleomycin induces A549 senescence.

(A) A549 were treated with various BLM concentrations for 3 d. SA-β-gal staining was applied to detect cellular senescence (original magnification, 400×). **(B, C)** The mRNA expression of aging-related markers (p16 and p21) was detected in A549 that was stimulated by various BLM concentrations for 3 d. **(D)** Western blot of aging-related markers (p16 and p21).

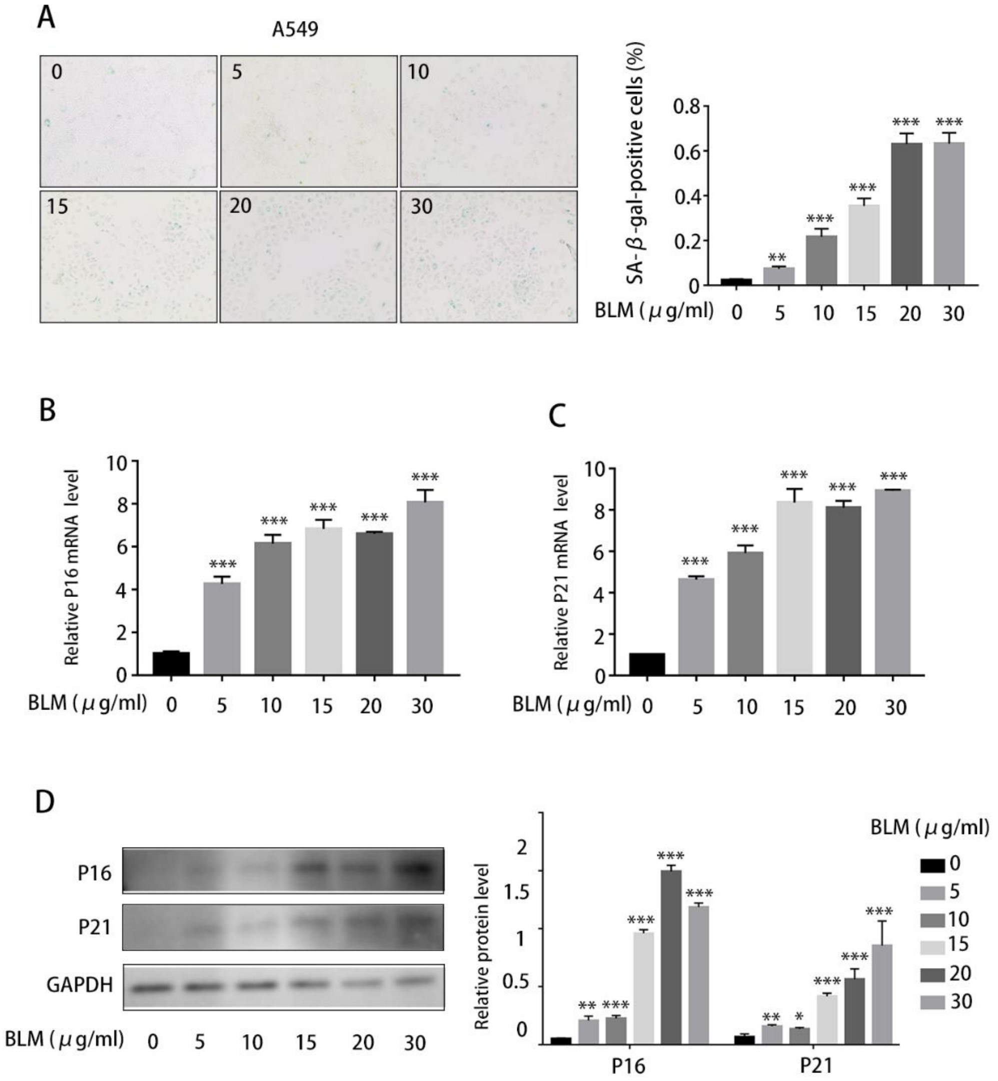

Figure S2. Representative pictures of HE staining, Masson's staining, and IHC staining (p21 and p16) of normal lung tissue and IPF lung tissue (original magnification, 200×).

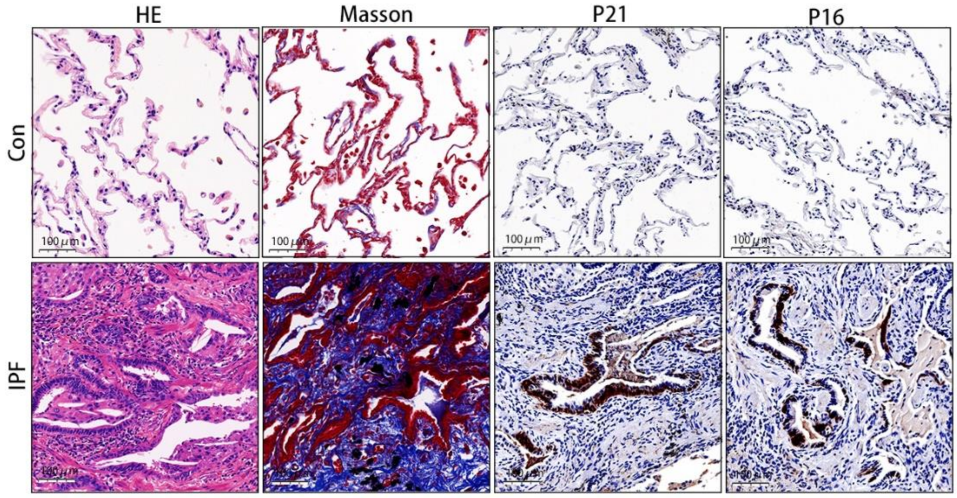

Supplementary Table 1: The information of the top 20 screened key genes between Fructus Arctii and IPF targets

Target name	Degree	Betweenness	Closeness
EGFR	32	525.9671	0.75
CASP3	28	187.70872	0.6666667
HSP90AA1	26	142.26202	0.6486486
MMP9	23	168.72021	0.6233766
ESR1	23	126.809044	0.61538464
ERBB2	23	81.69875	0.61538464
MDM2	19	31.325909	0.5925926
MAPK1	19	71.10856	0.6
MMP2	17	40.16694	0.57831323
AR	16	12.986918	0.55172414
PPARG	16	240.86961	0.5714286
MAPK14	16	32.210052	0.5714286
PTK2	15	39.398354	0.54545456
APP	15	115.237045	0.5714286
PARP1	14	46.74733	0.53333336
NR3C1	14	77.62062	0.5647059
MET	14	9.745526	0.53333336
CTSB	11	36.151897	0.52747256
TOP1	11	10.180686	0.5106383
ESR2	10	0.32051283	0.5106383

Supplementary Table 2: The molecular docking of ARC and target proteins.

Protein	Binding energy (kcal/mol)	Center			Size		
		x	y	z	x	y	z

EGFR	-7.6	3.379	-7.086	-0.742	30	30	30
MAPK1	-8.0	-2.990	5.786	12.400	30	30	30
PIK3CD	-6.5	39.582	13.893	33.832	30	30	30
MAPK14	-8.2	-9.739	-0.252	-12.694	22	22	22
MDM2	-6.4	12.380	14.249	3.675	30	30	30
ESR1	-7.6	-4.353	8.687	19.284	30	30	30
MMP1	-6.6	25.232	22.367	24.321	30	30	30
CXCR2	-6.8	-1.117	0.930	-7.627	30	30	30
